# The Binary Protein Interactome of *Treponema pallidum *– The Syphilis Spirochete

**DOI:** 10.1371/journal.pone.0002292

**Published:** 2008-05-28

**Authors:** Björn Titz, Seesandra V. Rajagopala, Johannes Goll, Roman Häuser, Matthew T. McKevitt, Timothy Palzkill, Peter Uetz

**Affiliations:** 1 Institute of Genetics, Forschungszentrum Karlsruhe, Karlsruhe, Germany; 2 The Institute of Genomic Research (TIGR) and J Craig Venter Institute (JCVI), Rockville, Maryland, United States of America; 3 Department of Molecular Virology and Microbiology, Baylor College of Medicine, Houston, Texas, Houston, United States of America; University of Liverpool, United Kingdom

## Abstract

Protein interaction networks shed light on the global organization of proteomes but can also place individual proteins into a functional context. If we know the function of bacterial proteins we will be able to understand how these species have adapted to diverse environments including many extreme habitats. Here we present the protein interaction network for the syphilis spirochete *Treponema pallidum* which encodes 1,039 proteins, 726 (or 70%) of which interact via 3,649 interactions as revealed by systematic yeast two-hybrid screens. A high-confidence subset of 991 interactions links 576 proteins. To derive further biological insights from our data, we constructed an integrated network of proteins involved in DNA metabolism. Combining our data with additional evidences, we provide improved annotations for at least 18 proteins (including TP0004, TP0050, and TP0183 which are suggested to be involved in DNA metabolism). We estimate that this “minimal” bacterium contains on the order of 3,000 protein interactions. Profiles of functional interconnections indicate that bacterial proteins interact more promiscuously than eukaryotic proteins, reflecting the non-compartmentalized structure of the bacterial cell. Using our high-confidence interactions, we also predict 417,329 homologous interactions (“interologs”) for 372 completely sequenced genomes and provide evidence that at least one third of them can be experimentally confirmed.

## Introduction

Most bacterial genomes encode hundreds or even thousands of proteins of unknown function [1]. If we want to understand the biology of these organisms, we need to understand the role of their proteins. One way to unravel the molecular function of a protein is to identify interacting proteins [2].

Up to now, the protein networks of only three organisms have been comprehensively investigated. Systematic purification of protein complexes and their identification by mass spectrometry has recently been completed in both budding yeast and *Escherichia coli* [3]–[5]. However, it became clear that these studies recovered only a fraction of all complexes and interactions [6] and it is still unclear how many interactions take place in a cell since no organism has been sampled exhaustively. More important, for the majority of interactions it remains unclear what their biological significance is.

Only recently, the first comprehensive bacterial yeast-two-hybrid (Y2H) interaction map was presented for *C. jejuni* [7]. Partial Y2H interaction maps have been published for human, fly, and worm [8] and for several bacteria including *Helicobacter pylori* [9], *Synechocystis* sp. [10] and *Mesorhizobium loti* [11]. Similar to purified complexes though, yeast two-hybrid data reveal only a fraction of all interactions with false negative rates estimated to be in the range of 50–90% [12]. Low coverage can only be overcome by applying multiple methods to the same organism [13] or studying homologous proteins in multiple organisms [14].

We have tested nearly all binary combinations among the proteins of ***Treponema pallidum***, the causative agent of syphilis, using the yeast two-hybrid system. With 1.14 Mbp and 1,039 ORFs [15], *T. pallidum* has one of the smallest genomes of any bacterium with an extracellular life-style. Although syphilis is usually not a life-threatening disease, it still caused 12 million new infections as recently as 1999, mostly in developing countries [16]. Progress in understanding the Syphilis disease and the biology of *T. pallidum* is severely hampered because *T. pallidum* cannot be cultured continuously *in vitro* and is not susceptible to genetic manipulation. However, our functional genomics studies demonstrate that insights into the function of individual proteins and larger functional complexes can be gained even for a bacterium which is not approachable by direct experiments. *T. pallidum* is only remotely related to other bacteria but still shares a significant fraction of conserved genes with other species [15]. Hence, we expect a substantial number of interactions to predict homologous counterparts in more tractable experimental systems as well as in other pathogens.

Given the significant false-positive rate in many Y2H screens it is necessary to verify these interactions by independent methods. In this study we have confirmed only 8 Y2H interactions for one simple reason: *Treponema pallidum* is not an experimentally tractable organism and thus it will remain difficult to investigate the biological relevance of these interactions. We suggest that interactions found in species such as *T. pallidum* be verified in more mainstream model organisms such as *E. coli*. We have previously shown the efficiency of such an approach for interactions among *T. pallidum* motility proteins by analyzing their homologous proteins and interactions in *E. coli* and *Bacillus subtilis* [14].

The aim of this study was to unravel the protein network of a single cell by means of the yeast two-hybrid system, evaluate its utility when compared to other experimental approaches and compare the resulting data to other genome-wide datasets. We conclude that the Y2H as used here may recover one quarter of all interactions and may require other methodologies or multi-species approaches to achieve a more complete coverage. Our dataset indicates for the first time that some operons can interact via their contained proteins and that bacterial cells exhibit more promiscuous interaction patterns than eukaryotic proteomes. We support the latter observation with data from yeast and speculate that this property is a consequence of the much less compartmentalized organization of prokaryotic cells when compared to eukaryotes.

## Results and Discussion

### Generation of a comprehensive binary protein-interaction map and quality control

**Figure 1:**
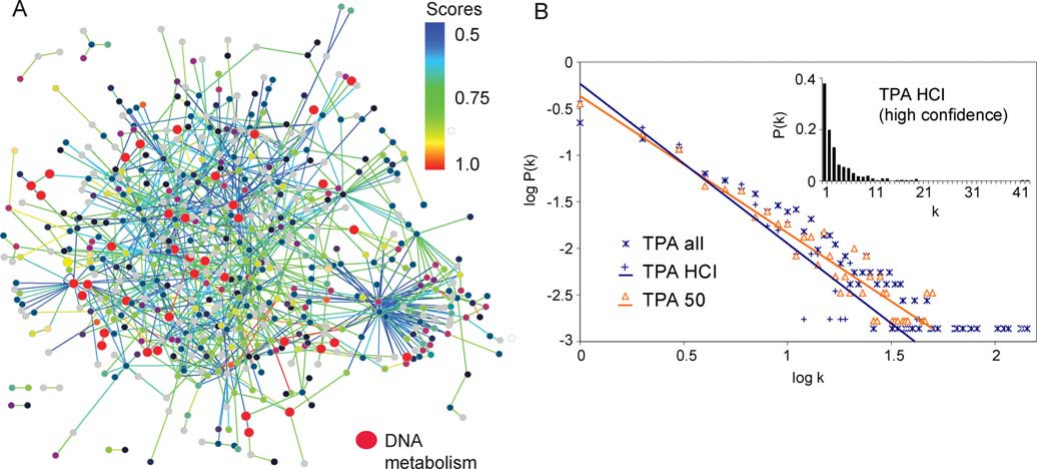
The protein interaction network of *T. pallidum* *.* A: High-confidence protein interaction network (TPA HCI 0.5) including 576 proteins and 991 interactions. Nodes are color-coded according to TIGR main roles. Links are color-coded based on their logistic regression score (indicated as spectral scale). Proteins involved in DNA metabolism (Figure 4) are shown as enlarged red circles. Note their distributed topology. See Table S1 for all interactions and scores. B. Comparison of the approximated power-law degree distributions of the *T. pallidum* networks. Node degrees k and their relative frequency P(k) are plotted on a bilogarithmic scale and fitted by linear regression. “TPA all”, “TPA 50”, and “TPA HCI” are the complete *T. pallidum* network and sub-networks filtered by “preycount” or logistic regression, respectively. The insert shows the node degree distribution of the high-confidence *T. pallidum* network (TPA HCI 0.5) on a linear scale.

Yeast-two-hybrid screening for the *T. pallidum* proteome was conducted in a systematic array-based format as described previously [14], [17]. In particular, the array format ensures reproducibility and control for unspecific background activation. Of nearly 1,000,000 examined protein pairs, 3,684 tested positive in our yeast two-hybrid assays resulting in 3,649 distinct interactions (Figure 1A, Table 1, Table S1). While we ranked all interactions based on various quality criteria, we decided to publish the whole dataset despite the fact that it may contain a significant number of false positives. We believe that this makes our results more transparent and also allows other researchers to investigate their own quality scoring algorithms.

**Table 1:** Topological properties of presented interaction networks.

	All	TP50	HCI0.3	HCI0.5	HCI.0.7
Filtering: in degree	-	<50	-	-	-
Filtering: log regr. score	-	-	>0.3	>0.5	>0.7
False negative rate (1−sensitivity)	-	-	18%	20%	50%
False positive rate (1−specificity)			52%	28%	12%
proteins	726	601	640	576	422
Interactions in directed networks^1^	3684	1634	1628	992	414
Av. Node degree	10	5.4	5	3.4	1.9
Av. Shortest path length	2.95	3.88	3.95	4.73	8.08
Power coefficient	1.15	1.47	1.54	1.71	2.35
R^2^	0.85	0.91	0.91	0.87	0.94

1 (includes reciprocal interactions).

Topological parameters for *T. pallidum* protein datasets and corresponding networks were calculated using the NetAnalyzer plugin for Cytoscape (http://med.bioinf.mpi-inf.mpg.de/netanalyzer/): whole network “all”, network filtered by in-degree “TPA 50”, and networks filtered by logistic regression score “HCI 0.3” - “HCI 0.7”. In addition, the false negative and the false positive rates after 10× cross validation are given for the datasets filtered by logistic regression.

We used two independent approaches to derive more reliable, “high-confidence” datasets from our raw two-hybrid data: first, a simple approach based on the number of times a certain protein is found as prey: preys which are found more than 50 times (which is an arbitrary threshold) are likely to be unspecific interactors and thus have been excluded from the “TPA 50” dataset. Second, we applied a more comprehensive logistic regression approach, similar to that used in the STRING database [18](high-confidence dataset, see methods for details). In the latter high-confidence network (“TPA HCI”), 576 proteins of *T. pallidum* are connected by 991 distinct interactions with an average of 3.4 interactions per protein. Based on our training dataset, the false positive rate of this set can be estimated to be 28% (see Supporting online information [SI] file [Discussion S1] for details). However, since there are no objective computational ways to unambiguously identify false positives or negatives in any interaction dataset further experimental verifications are required for better assessments. Table 1 and Figure 1B show a summary of the topological properties of the network.

### Comparison of datasets

Up to now, only two comprehensive studies of protein interactions in bacteria have been published [5], [7]. In addition, a number of partial prokaryotic interaction studies have been presented, including Y2H maps [9]–[11] and another coAP/MS study for *E. coli* [19]. Surprisingly, only 26 *T. pallidum* interactions were shared with *C. jejuni*, only 23 interactions with *E. coli*, and only 5 with *H. pylori* (Table S1). While the small overlap seems to be surprisingly low, small overlaps between interaction datasets are commonly observed, and may be explained by the large phylogenetic distance between these species, the different methodologies applied, the considerable false negative rate, and the incomplete sampling of each interactome.

### Total number of interactions of a minimal bacterial proteome

To estimate the overall false-negative rate of our Y2H screen, we made use of a comprehensive set of flagellar protein interactions, which we collected for a comprehensive study on bacterial motility [14]. In this study, a “gold standard” dataset of 59 motility interactions was used, of which 39 had homologous pairs in *T. pallidum*. Of these 39 pairs, only 9 (or 23%) were found in our dataset which would imply a false-negative rate of 77% (but see below). To estimate the false positive rate, we looked for ‘high-confidence’ interactions which were maximally separated in a network of protein families (StringDB experimental COG network - exp. score>0.15). Based on the overlap, we estimate the false positive rate of our high-confidence set to be 28%. Based on our high-confidence set with 991 interactions, we can predict a total number of approx. 3,100 interactions (total interactions = found interactions−false positives+false negatives) for *T. pallidum* with an average of ∼6 interactions per protein.

Large-scale interaction studies cover functional complexes only to a limited extend. Integration of several datasets is the first choice to increase the coverage as has been recently demonstrated by our group for bacterial motility where a combination of two-hybrid data from *T. pallidum* and *C. jejuni* reduced the false-negative rate from 77% and 87%, respectively, to a combined 67% [14]. We expect that further technical improvements and the addition of even more genomes may be able to reduce the false negative rate to below 50%.

### Mapping of the interactome onto the genome

**Figure 2:**
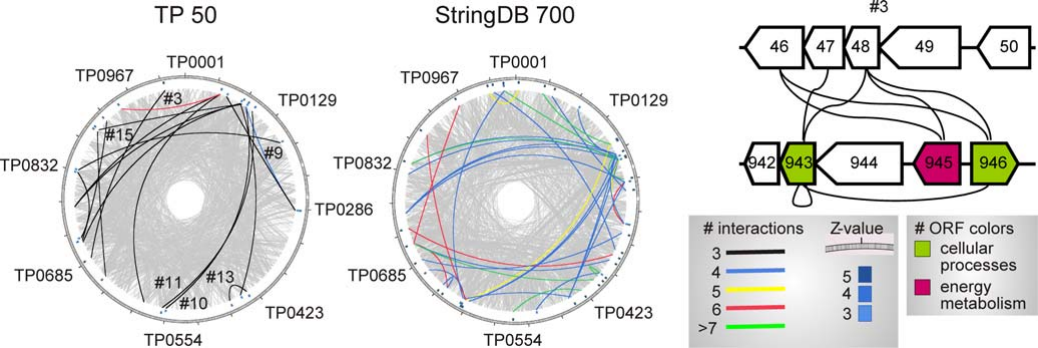
Genomic locations linked by protein interactions. A,B. Certain genomic locations are especially tightly linked via protein interactions when compared to randomized networks. Genomic location links are visualized for the “TPA 50” protein interaction dataset (A) and for bioinformatical associations from the String database (B, “StringDB 700”, protein links with combined score>0.7) [18]. Grey lines indicate all individual protein interactions/associations connecting genes on the circular chromosome of *T. pallidum* (1.14 Mbp total size). Tightly connected clusters comprising 5 or more neighbouring genes were identified (thick lines) by a computational method, which is based on the comparison to re-wired versions of the network (see methods). The number of linking interactions between two clusters is indicated by the color of their connecting line and the enrichment compared to randomly re-wired networks is indicated by a Z-value (in the outer circle at the positions of the clusters). Due to the incorporation of genomic neighbourhood links by the String database (and for clarity), self-links between genomic locations are removed in the “StringDB 700” representation. C, The region flanking FliS (TP0943) is, for example, connected to the region of TP0046–TP0048, linking motility and sugar metabolism (TP0943–TP0946) to a cluster of uncharacterized proteins around TP0047 which appears to be involved in motility as well [14].

On the genome level, bacterial genes have long been known to be organized in functional groups such as operons or as co-conserved genomic islands [20]. Many structural features of interactomes have been revealed including the tight connection of functional protein complexes (e.g., [21]). We wondered, whether an interdependence of the genome and the interactome structure could be identified. To this end, we overlaid Y2H interactions and predicted gene associations [18] onto the circular *T. pallidum* chromosome (Figure 2). The overlay shows that all regions of the circular chromosome are highly connected both by Y2H interactions and by the predicted functional connections clearly indicating that the given genome structure does not constrain the overall flexibility of physical interconnections. Despite this overall tendency, we wondered whether especially tightly connected pairs of genomic loci are present. In other words, we were looking for operons or “neighborhoods” of which multiple proteins interacted with multiple proteins from operons or neighborhoods elsewhere in the genome. For this, we applied a filtering algorithm (which involves the comparison with randomized networks) to enrich highly connected genomic loci (Figure 2, Table S2). As anticipated, many interactions connected genomic loci of well known protein complexes such as the ribosome (8 links) or the bacterial flagellum (5 links). One striking example in the TPA50 data set is link #3 involving six proteins and six interactions which connect the region flanking FliS (TP0943), the flagellin chaperone, and the region of the uncharacterized proteins TP0046–TP0048 (Figure 2). In addition to FliS, TP0046 and TP0048 have also been functionally implicated in bacterial motility and the ortholog of TP0945 shows a motility phenotype in *E. coli* [14]. This suggests that the locus around TP0048 has a functional involvement in bacterial motility, and demonstrates that genomic loci links can have functional implications.

### Functional class organisation

**Figure 3:**
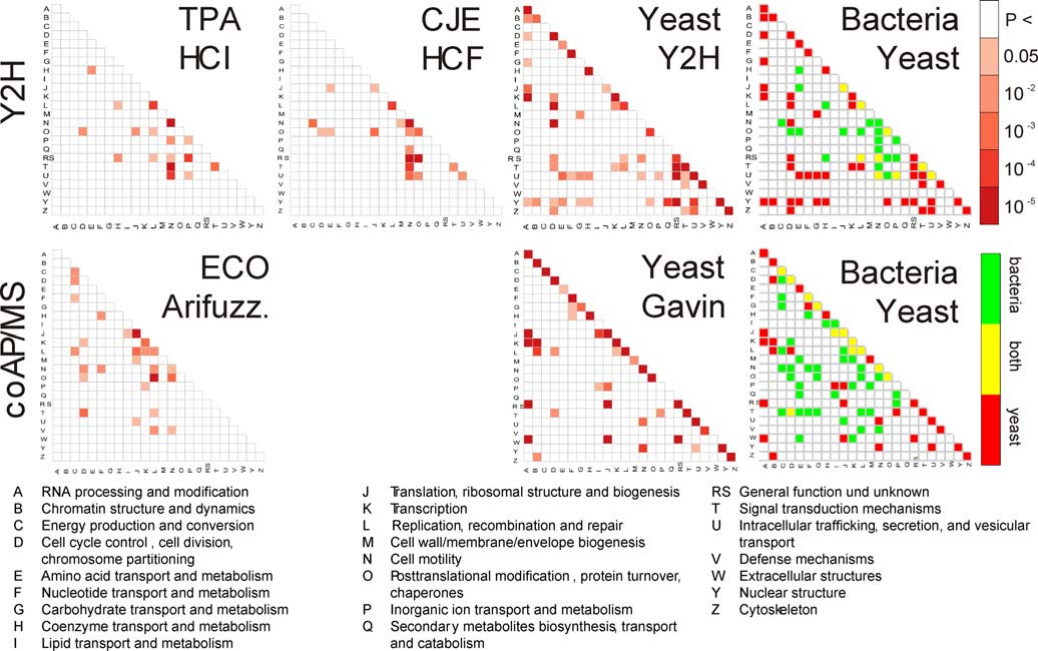
Interactions between functional classes in pro- and eukaryotes. Connections between functional classes mediated by protein-interactions in Y2H datasets (TPA HCI = *T. pallidum* high confidence interactions, CJE HCI = *Campylobacter jejuni* high confidence interactions), and two comprehensive coAP/MS datasets from *E. coli* [5] and yeast [3]). For each data set and each class combination, a functional class association index (fCAI) was calculated (see methods), which scores the interaction density between two functional classes in a dataset of given size and class coverage. The matrices show the significance of each enriched functional class link (see color key). Results obtained from genome-wide Y2H (top) or coAP/MS (bottom) experiments are compared between bacteria and yeast (see color key).

The main difference between pro- and eukaryotes is their subcellular organization. We wondered whether this functional specialization is reflected in protein interaction networks. To investigate this, we grouped all proteins belonging to the same functional category (as defined by the STRING database [18]) and counted the links within these groups and between groups. Figure 3 shows functional link matrices for several large-scale interaction datasets, including the *Treponema* network. Surprisingly, on the level of interactions between functional groups, prokaryotic datasets appear to be more similar among each other than eukaryotic datasets (Figure 3). That is, in bacteria proteins seem to have more interactions with functionally unrelated proteins than eukaryotes do. Interestingly, this observation cannot be an artifact of the yeast two-hybrid system as the same pattern can be seen in protein complex purification data from *E. coli* [5] and yeast [3], [4]. This comparison also reveals cross-talk between different processes, e.g. that the “cell motility” class occupies a central position in prokaryotes: while proteins in this class interact mostly with themselves, they also have multiple links to the “signal transduction mechanisms”, the “secretion”, and to the “energy production” classes.

**Figure 4:**
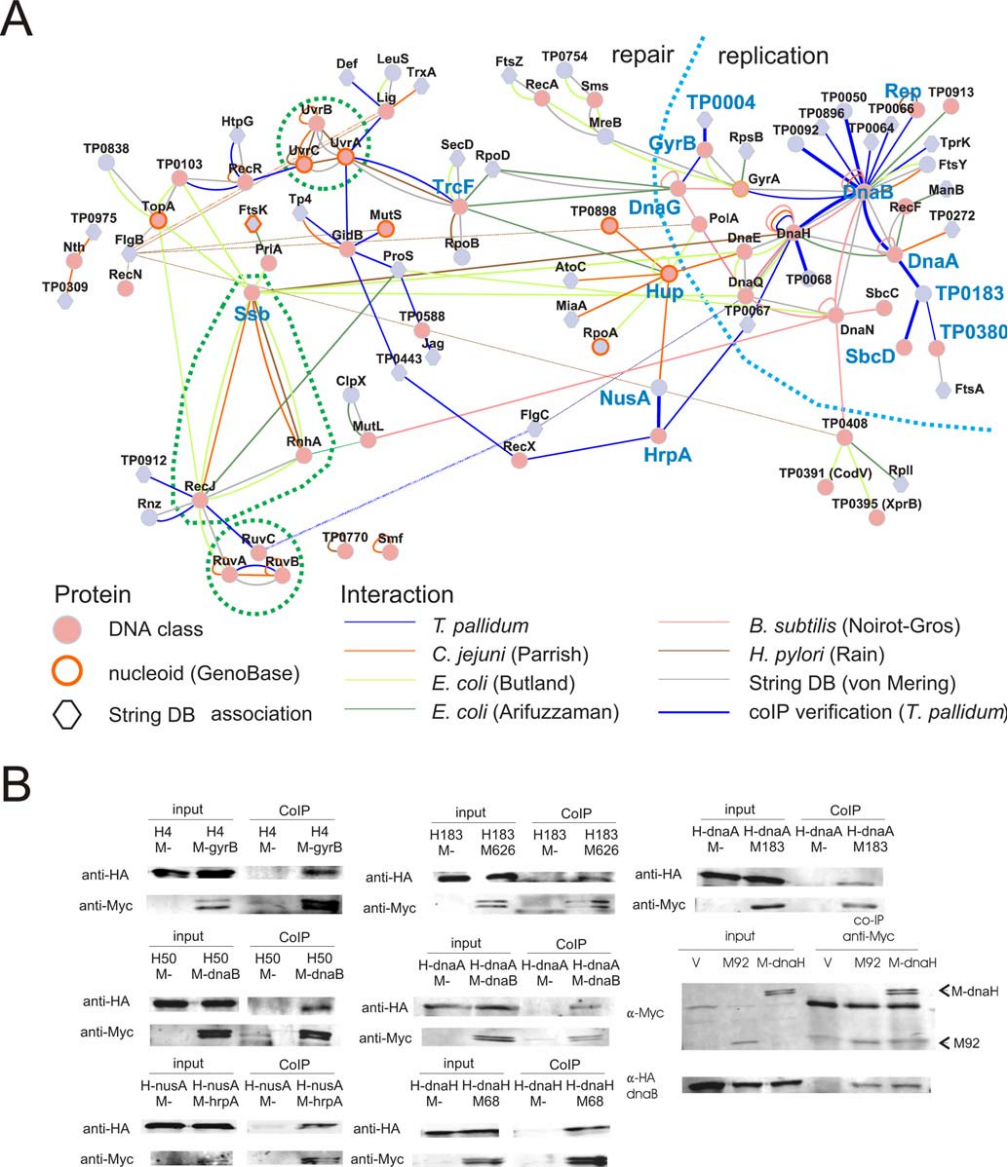
An expanded view on DNA metabolism. A. The DNA metabolism network for *T. pallidum* based on the integration of several experimental and bioinformatical data sets (see methods). *T. pallidum* proteins with a DNA metabolism related function (red nodes) are linked by interactions from several high-confidence protein interaction datasets. The color of the interactions indicates their source (see color key), e.g., all blue interactions were identified in our study (i.e. in *T. pallidum*) and are part of the high-confidence interaction dataset for *T. pallidum* (for detailed list see Table S3b). Proteins of other functional classes are included, when their association is supported by at least one additional evidence. Grey lines indicate support of an interaction by bioinformatical predictions (String database score>0.4). Proteins with orange borders have been shown to localize to the nucleoid. Proteins with a hexagonal shape have a tight bioinformatical link to a DNA metabolism protein (String database score>0.8). Proteins that are discussed in the text are shown in larger, blue font. B. Co-immunoprecipiation (coIP) experiments for a number of selected DNA metabolism interactions are shown (thick lines in network). The coIP is conducted with an anti-Myc antibody. For each coIP, the total input and the fractions after coIP are analyzed by Western Blot probing with an anti-HA and an anti-Myc antibody as indicated on the left of each blot. The empty Myc-tag vector “M-” is used as a control for unspecific binding of the HA-tagged protein. HA-tagged proteins are labeled with “H” and their gene name or gene number, e.g. “H4” in the first coIP corresponds to HA-tagged protein TP0004. Accordingly, Myc-tagged proteins are labeled with “M”, e.g. M-gyrB corresponds to Myc-tagged GyrB protein.

The number of self-links, i.e. functional links on the diagonal of the matrix, can be assumed to give an indication of the functional organization in a dataset or a species. We noticed that the number of self-links is larger in eukaryotes than in prokaryotes: *T. pallidum* (4 links, 1,039 genes), *C. jejuni* (6 links, 1,654 genes), *E. coli* (7 links [the average between [5] and [19]], 4,289 genes), yeast (16 links [Y2H], 20 links [Gavin], 6,200 genes). One explanation for these differences could be the source of the data: coAP/MS approaches tend to favor stable complexes and proteins within the same complex are usually assigned to the same functional class. On the other hand, Y2H favors transient interactions [13] among proteins which may be more promiscuous and thus less-well defined functionally. An alternative explanation for the differences in functional linkage is an increase in functional complexity from bacteria to eukaryotes, with a fundamental difference in the functional organization between these cell types. A slightly higher number of self-links in the yeast coAP/MS dataset compared to the combined yeast Y2H dataset argues for the former explanation. Comparing the three datasets of bacterial origin with the yeast datasets (coAP/MS and Y2H), however, supports an alternative explanation: a higher level of functional organization is observed in the eukaryotic datasets. Thus, the well-known difference in structural organization of pro- and eukaryotes is also reflected on the protein-interaction level. Functional processes are well separated in eukaryotes, e.g., through differential compartments such as organelles, whereas most functional processes in prokaryotes co-exist in space and partly in time as exemplified by the synchronous execution of transcription and DNA replication. It remains to be seen whether these results can be generalized for more species when additional datasets for other prokaryotes and eukaryotes become available.

### An integrated view of DNA-metabolism related processes

In addition to the protein network of bacterial motility [14], we here present an additional network of DNA metabolism for *T. pallidum,* which is solely based on high-throughput data and bioinformatical predictions (Figure 4A, see Discussion S1 and Table S3 for an extended discussion). For additional support, a number of interesting interactions were validated by co-immunoprecipitation (Figure 4B). Several known functional complexes can be identified including the Uvr-system, the Ruv-system, and the DNA replication complex. DNA replication and DNA repair are linked, e.g. by the transcription-repair-coupling factor (TrcF), which links stalled RNA polymerases to nucleotide excision repair [22]. Here, TrcF is linked to DNA replication via the DNA primase (DnaG), and thus could participate in the clearance of stalled RNA polymerase arrays, which eventually block DNA replication in prokaryotic cells [23]. As anticipated, two DNA binding proteins, Ssb (single-stranded-DNA binding protein) and Hup (HU protein), with ubiquitous functions occupy a central position in the network [24]–[29]. The RNA helicase HrpA interacts with the (anti-) termination protein NusA indicating an involvement of HrpA in this regulatory process. The single-stranded DNA dependent helicase Rep is required for genome replication [30] and acts with PriC in the replication fork restart pathway. However, its specific role is unknown [31]. The direct interaction of Rep with the replication helicase DnaB could indicate a concerted action of these helicases during replication fork restart. In addition, several proteins were newly associated with DNA-metabolism including a number of *Treponema* or *Spirochete* specific proteins (Table 2). The protein TP0004, for example, interacts with GyrB of the gyrase complex. Since TP0004 is located in a conserved gene cluster together with GyrA (TP0005) in spirochetes, a functional connection of TP0004 with the gyrase is very likely. The *Treponema* specific protein TP0183 interacts with the DNA replication initiation protein DnaA, with SbcD, which removes DNA hairpin structures that lead to stalling of DNA replication [32], and a DNA repair helicase TP0380. Thus, TP0183 might help to reinitiate DNA replication after DNA repair mediated by SbcD and TP0380. All in all, the DNA metabolism network mirrors biological knowledge from small-scale experiments, and demonstrates the potential of these approaches to uncover novel biological insights.

**Table 2:** Novel functional assignments based on protein network and additional evidence.

Gene	Novel Function	Evidences
TP0004	Gyrase associated protein	GT (gyrase, gyrA); PI (gyrase, gyrB)
TP0050	DNA replication, nucleotide metabolism	PI (dnaB), DOM (phophoribosyl transferase)
TP0064, TP0066, TP0067, TP0068	Operon involved in DNA metabolism (+ cell division)	PI (DNA metabolism + cell division); GBAA (TP0066, cell division/chromosome partitioning); DOM (TP0065, DNA methylase); HOM (TP0067, putative cell div. protein)
TP0183	DNA metabolism	GBAA (DNA metabolism); PI (dnaA, sbcD, DNA repair helicase)
TP0297	Cell wall metabolism	PI (capsular polysaccharide biosynthesis protein); DOM: (SPOR = involved in peptidoglycane binding)
TP0320 (a)	dsDNA and nucleotide uptake	PI (ribulose-3-P-epimerase & exonuclease for dsDNA); GT (TP0319, TP0322, and TP0323 [rib/gal transporter])
TP0443	DNA metabolism and/or repair	PI (gidB (tRNA methyltransferase), recX); DOM (DALR anticodon binding domain); GT (recN)
TP0496	Membrane protein involved in translational and cell division	PI (tRNA-synthetases, DNA primase); GBAA (translation); GT (rod-shape determining proteins)
TP0526 (b)	transcription termination/antitermination	PI (nusA)
TP0561 (c)	Membrane protein chaperone	PI (with membrane proteins), DOM (SsgA, sporulation, cell division)
TP0580 (e)	ABC transporter, polysaccharide (antigen) synthesis (dTMP sugar)	PI (uridylate kinase) (enzyme complex); DOM (GtrA): generation of sugar building block
TP0650	Translation	GBAA (translation); GT (tRNA-synthetases); PI (peptide deformylase; ribosomal protein L32)
TP0658 (f)	Flagellar assembly factor fliW	PI (flagellin); GT (motility)
TP0772	Transcription Regulator	PI (RNA-polymerase, TP0701); HOM (LysR family transciptional regulator, KEGG, SW-Score 122)
TP0920	Energy production	GBAA (energy); GT (Oxidoreductase, TP0921)
TP0941	Regulation of motility	GBAA (signal transduction); PT (FlgM); GT (FliS, FlgN)
TP0963 (d)	ABC transporter, membrane biogenesis	PI (TP0965); DOM (FtsX); GT (ABC transp., lipoprotein metabolism)

All proteins in this table are currently annotated as (conserved) hypothetical [33]. Used evidence codes are: PI (protein interaction), GT (genomic context), DOM (protein domain), genomic loci link (GLL), guilt-by-association approach (GBAA), homology (HOM). **Notes and references**: **(a)** TP0319 is a purine nucleotide receptor and its whole operon probably involved in nucleotide import [41]; **(b)** ATP-dependent helicase (HrpA). **(c)** SsgA like proteins play a chaperonin-like role [42]; **(d)** Transporter complex with TP0965 (HlyD motif). (e) See ref. [43], (f) See ref. [34].

### Functions of unknown proteins

In total, 433 proteins of *T. pallidum* (42% of the proteome) are still uncharacterized [33]. Thus, we expanded our interaction-based annotation from DNA metabolism to the whole dataset. Indeed, 649 out of the 991 interactions in the high-confidence set involved at least one uncharacterized protein. 493 of these interactions link an uncharacterized protein to a protein of known function. These protein-pairs can be used to derive improved annotations, e.g. by integrating datasets for specific functional groups such as DNA metabolism (Figure 4) or bacterial motility [14]. More typically, a bioinformatical guilt-by-association approach and manual curation of highly-reliable interactions are used. A selection of 18 new annotations derived from our data is given in Table 2. These (initial) annotations form an in-route for further characterization. As an example, we recently characterized the protein TP0658 as a novel bacterial assembly factor based on its interaction with flagellin proteins [34].

### Patterns of conserved interactions

**Figure 5:**
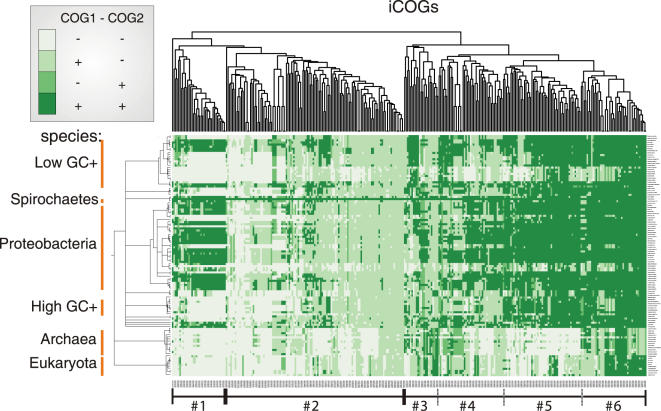
Interacting clusters of orthologous groups (“iCOG”) show phylogenetically conserved interaction patterns. Each row of the shown profile corresponds to a species and each column corresponds to a pair of interacting protein families (i.e. iCOG), for which an interaction was found in the high-confidence *T. pallidum* data set. The protein families were defined based on the “cluster of orthologous genes” approach (COG) (see methods). With this, the profile shows for each interaction of the *T. pallidum* data set whether both interacting proteins, only one interacting protein or none of the interacting proteins are conserved in a given species (given row). For each species from the shown taxonomy (y-axis) and each iCOG, a conservation value is shown in the matrix. This conservation value indicates whether both COGs are conserved/absent in a given species or whether only one or the other COG is conserved (see left upper corner for color key). Overall, three distinct conservation regions are visible in the clustered matrix: #1, #2, and region #3-#6, which we subdivided somewhat arbitrarily into individual clusters #3-#6 with increasing conservation from left to right (note branches on tree above). This figure is also available as zoomable Figure S1 in PDF format in which individual species names and iCOGs can be seen.

Out of 1,039 *T. pallidum* genes, 302 are Spirochete-specific and an additional set of 147 genes shows a “narrow” distribution and is conserved in less than 50% of the sequenced bacterial species. Interestingly, a majority of 758 (76%) *T. pallidum* interactions (HCI) involve at least one of the 449 “narrowly” distributed proteins. Based on this observation, we asked how the overall distribution for interacting proteins looks like. For this, we constructed a phylogenetic profile for interacting protein families (“iCOGs”, Figure 5). These profiles could be separated into distinct conservation clusters by a matrix clustering approach. The most striking pattern is observed in cluster #1, in which the interacting proteins are either both absent or both present in a given species. This cluster is highly enriched for motility-related interactions (35 fold enrichment, p = 1.1×10^−20^), which explains the observed pattern by the distribution among motile bacteria. Cluster #6 shows the highest conservation and is enriched in translation-related functions in archaea and eukaryotes (cluster #6, 5 fold enrichment, p = 3.9×10^−7^). On the contrary, the large cluster #2 contains mainly *Treponema* or *Spirochete* specific proteins, which interact with broadly conserved proteins, and is enriched for proteins of unknown or general function (3 fold, p = 0.003). For *Spirochete*-specific proteins, we also find a general tendency to interact with well-conserved proteins, which are conserved in 60%–80% (z-score vs. random of 2.0) or in 80%–100% (z-score of 1.1) of the sequenced species. Despite the large number of *Spirochete*-specific proteins, their overall tendency to interact with well-conserved proteins supports the notion that specific properties of spirochetes (e.g., their endoflagella) have not been invented independently in evolution but rather derived by modification of existing structures or by recruiting spirochete-specific proteins.

### Prediction of protein interactions in other species

**Figure 6:**
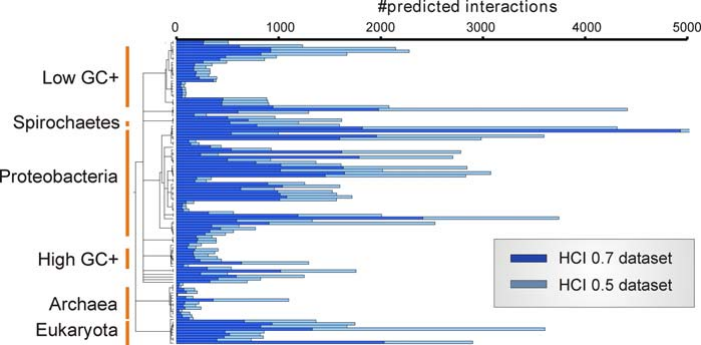
Prediction of interactions for other species based on *T. pallidum* high-confidence data sets. Species (y-axis) are ordered according to taxonomy (broad groups are indicated) and the number of predicted interactions for each species based on two confidence score cut-offs (HCI 0.5 with score>0.5 and HCI 0.7 with score>0.7) is shown.

Interactions in *Treponema* are likely to be conserved in other species. In fact, we have tested 174 motility-related interactions among *Campylobacter jejuni* proteins predicted from our dataset [14]. Using the criteria of Parrish et al. [7], 49 of those were tested positive with high confidence. Interestingly, most of them were not found in the study by Parrish et al. because their screens used pooled clones while our retests used individual clones. Pooling often results in lost interactions for poorly understood reasons. In any case, the comparison of *Treponema* and *Campylobacter* data confirms other studies where interactions predicted from yeast were also found in worm [35] or where metazoan interactions successfully predicted homologous interactions in yeast [36]. As a basis for further functional analysis and comparative interactomics, we predict 417,329 interactions in 372 other genomes (Table S4, Figure 6, and SI file “Data S1”). Based on our successful prediction of *Campylobacter* interactions using *Treponema* data, we estimate that about 118,000 (49/174*417,329) of these predicted interactions will turn out to be reproducible.

### Conclusions

Here we presented a genome-wide protein interaction map for *Treponema pallidum*, the causative agent of Syphilis. The genome of *T. pallidum* is one of the smallest of all bacteria not living within host cells, and most importantly, *T. pallidum* is not approachable by many experimental methods, since it cannot be cultured continuously *in vitro*. From its interaction map, we obtain insights into the connection between genomes and interactomes, we see that the different structural organization of pro- and eukaryotes is already reflected on the interaction level, and demonstrate the usefulness of our interaction data to reveal biological insights into biological processes (DNA metabolism) as well as into the function of individual proteins (e.g., HrpA). We learned that *Spirochete* and *Treponema-*specific proteins interact with ubiquitously conserved proteins and potentially modulate their functions to achieve *Spirochete*-specific properties. Finally, based on our high-confidence interaction data 417,329 interactions for 372 species can be predicted.

The biological relevance of the interactions found in this study remains to be shown in model organisms that are more tractable experimentally. Nevertheless, we believe in the utility of data obtained in organisms such as *T. pallidum* as they can show us which proteins and interactions are conserved in other species and thus help us to define minimal or essential sets of protein activities.

### Outlook

Protein interaction mapping is where genome sequencing was about 10 years ago. Many more interaction datasets are required to distinguish between conserved and non-conserved (but biologically relevant) interactions and separate them from false positives and false negatives. Such a classification will make it much easier to evaluate the biological significance of individual interactions, either by suggesting additional experiments or by facilitating computational analysis such as protein docking.

## Materials and Methods

Description of datasets and a more extensive description of the applied methods can be found as supporting information (Discussion S1). The interactions of this study have been submitted to the IntAct database (http://www.ebi.ac.uk/intact/, accession number EBI-1581350) and to the IMEx consortium (http://imex.sourceforge.net) through the MPIDB database (http://www.jcvi.org/mpidb, identifier IM-9152).

### Cloning of baits and preys, Y2H screening

The ORF clones from McKevitt et al. [37] were transferred into compatible bait and prey vectors pAS1-loxP, pLP-GBKT7Amp, and pLP-GADT7 [Clontech], by Cre-mediated homologous recombination. After transformation into yeast, all preys were arrayed and screened as described in [14].

### Selection of high-confidence datasets and logistic regression model for quality scoring

For the “TPA 50” dataset, preys that were found in more than 50 screens were removed as large numbers indicate unspecific interactions [14]. Based on a binary logistic regression model [18], we assigned probability scores to all interactions using a training set of positive (interologs in DIP and IntAct) and negative *Treponema* interactions (see Discussion S1 for more details on the training data and scoring procedure). Next, we generated a set of ‘highly reliable interactions’ (TPA HCI) retaining only those with a probability > = 0.5. At this probability cutoff, 80% of interactions in the positive training set are classified correctly (true positives), while 28% of negative interactions were misclassified (false positives).

### Links between genomic locations (Figure 2)

The number of interactions or bioinformatical associations between clusters of five neighboring genes was counted for the real network and for randomized versions of this network. Overrepresentation of a link compared to 1000 randomized networks was assessed by calculating a Z-score, 
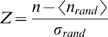
, with the number of linking interactions n, its average in 1,000 randomized networks 〈n_rand_〉, and its standard deviation σ_rand_. Links between gene clusters with at least three connecting interactions/associations and a z-score (compared to random networks) of at least 2 are shown in Figure 2.

### Associations of functional classes (Figure 3)

Association values were calculated for the functional classification scheme of the String database [18]. First, the functional class association index (fCAI) was computed for each dataset and each functional class pair. The fCAI represents a log-odds-ratio, which compares the odds to find the number of linking interactions in the experimental set to the odds in a random model (see discussion S1). Based on a z-statistic, a raw p-value was derived for each functional class link and used for the visualization of functional links in the association matrix.

### Extended view of *T. pallidum's* DNA metabolism (Figure 4)

A set of *T. pallidum* proteins involved in DNA metabolism was extracted from several databases (Table S3). Several interaction sets were integrated: high-confidence *T. pallidum* Y2H set (TPA HCI), high-confidence *C. jejuni* Y2H set [7], two socio-affinity-index (SAI) filtered *E. coli* coAP/MS sets ([19] and [5]), a *B. subtilis* Y2H set [38], a *H. pylori* Y2H set [9], and bioinformatically predicted interactions [18]. *E. coli* proteins localized to the bacterial nucleoid were taken from the GenoBase database (http://ecoli.naist.jp/GB6/search.jsp). The transfer of interactions between species (interologs) was based on orthology relationships from the MBGD database [39]. All *T. pallidum* interactions and interologs linking two DNA metabolism related proteins were selected. In addition, interactions or interologs of DNA metabolism related proteins, which were supported by bioinformatical predictions [18] (combined score>400) or by at least two experimental datasets, were chosen. Finally, associated proteins, which were predicted to be involved in DNA metabolism [18] (combined score>800 for DNA metabolism related proteins), localized to the nucleoid in *E. coli*, or had an additional evidence associated with it (Table 2) were included. Network visualization was done with the Cytoscape software [40]. A number of these selected interactions were re-tested by co-immunoprecipitation as described in [14] (Figure 4B). Briefly, *E. coli* BL21/DE3 cells were co-transformed with expression vectors carrying Myc-tag (vector pBad24Myc_loxP) and HA-tag (vector pBad33HA_loxP) fusions of the proteins to be tested. Protein expression was induced with 0.2% (w/v) L-Ara for 3 h at 37°C. The co-immunoprecipitation was performed with anti-Myc antibodies (Santa Cruz).

### Conservation Classes and iCOGs (Figure 5)

A matrix showing the conservation of iCOGs (interacting clusters of orthologous groups) in the “TPA HCI” data set was created. For each interaction in the interaction data set, an iCOG was defined, if both interacting proteins were part of a COG (cluster of orthologous group–meaning that they could be grouped with proteins from other species into an orthologous protein family). Each element of the matrix, contains a conservation value for a specific iCOG in a specific genome (species). The conservation value (cv) indicates whether both COGs of the iCOG are conserved (cv = 1) or absent (cv = 0) in the given species or whether only one or the other COG is conserved (cv = 0.5). Average linkage clustering of the matrix in iCOG direction was done with the R-package using Euclidean distances.

Significant enrichment of functional classes (taken from the STRING database) in the conservation clusters were identified employing Fisher's exact test in conjunction with a Bonferroni correction for multiple testing (p<0.01) using the R-package.

## Supporting Information

Table S1 All protein-protein interactions of Treponema pallidum found in this study.
(1.82 MB XLS)

Table S2 Additional genomic links as shown in Figure 2.
(0.11 MB XLS)

Table S3 All proteins involved in DNA metabolism as well as their interactions as shown in Figure 4.
(0.07 MB XLS)

Table S4 Summary table for the predicted interactions showing all species, their phylogenetic relationships, and the number of predicted interactions for each species.
(2.00 MB XLS)

Discussion S1 More detailed discussion of results and additional details on the methodology used in this study.
(0.42 MB DOC)

Data S1 Predicted protein-protein interactions based on our Treponema pallidum data; zip archive containing 372 files with one file per species.
(6.09 MB ZIP)

Figure S1 Interacting clusters of orthologous groups (“iCOG”) show phylogenetically conserved interaction patterns. Each row of the shown profile corresponds to a species and each column corresponds to a pair of interacting protein families (i.e. iCOG), for which an interaction was found in the high-confidence T. pallidum data set. The protein families were defined based on the “cluster of orthologous genes” approach (COG) (see methods). With this, the profile shows for each interaction of the T. pallidum data set whether both interacting proteins, only one interacting protein or none of the interacting proteins are conserved in a given species (given row). For each species from the shown taxonomy (y-axis) and each iCOG, a conservation value is shown in the matrix. This conservation value indicates whether both COGs are conserved/absent in a given species or whether only one or the other COG is conserved (see left upper corner for color key). Overall, three distinct conservation regions are visible in the clustered matrix: #1, #2, and region #3-#6, which we subdivided somewhat arbitrarily into individual clusters #3-#6 with increasing conservation from left to right (note branches on tree above).
(0.14 MB PDF)
