# Supplementary material for: The Binary Protein Interactome of Treponema pallidum – The Syphilis Spirochete
Source: PLoS One. 2008 May 28;3(5):e2292. doi: 10.1371/journal.pone.0002292 (PMC2386257; doi:10.1371/journal.pone.0002292)
Supplement: Discussion S1 — More detailed discussion of results and additional details on the methodology used in this study. (0.42 MB DOC) [file pone.0002292.s005.doc]

Titz et al., The protein interactome of *Treponema pallidum*.

Extended Discussion and Materials & Methods

Results and Discussion

**An integrated view of DNA-metabolism related processes (Figure 4):** Each large-scale interaction study covers a given functional complex only to a limited extend. This is due to the high false-negative rate of the employed method, which can be as high as 96% for Y2H (1) and which we have estimated to be in the range of 77% in our study. Integration of several datasets is the first choice to increase the coverage as has been recently demonstrated by our group (2). Here, we present an integrated network of DNA metabolism for *T. pallidum,* which is solely based on high-throughput data and bioinformatical predictions (Figure 4A). For additional support, a number of interesting interactions were validated by co-immunoprecipitation (Figure 4B). Several known functional complexes can be identified including the Uvr-system (involved in base-excision repair), the Ruv-system (resolves recombination junctions), and the DNA replication complex (including DnaA/B, DNA polymerase, and gyrase). The DNA replication proteins can be roughly separated from the DNA repair proteins. However, several points of contact between these processes are visible. Transcription-repair-coupling factor (TrcF) links stalled RNA polymerases to nucleotide excision repair (3). In addition to this known connection, TrcF appears in a protein complex with the DNA primase (DnaG), which implies an involvement of TrcF in DNA replication. In this respect, TrcF could clear stalled RNA polymerase arrays, which eventually block DNA replication in the prokaryotic cell, which does not show temporal separation of transcription and DNA replication (4). The majority of additional links between replication and repair are formed by two DNA-binding protein: the single-stranded-DNA binding protein (Ssb) and the HU-protein (Hup). This linking function is anticipated, since both proteins are involved in multiple DNA-related processes. Ssb is involved several processes such as DNA supercoiling/catenation (5), recombination initiation (6), and DNA unwinding (7, 8). The HU-protein is involved in several processes, such as recombination/repair and DNA supercoiling (9, 10). Many of the indicated functions of these proteins are already suggested by their protein interactions shown in the network.

Several previously unrecognized connections are also visible in the network. Interestingly, in several datasets, the replication helicase DnaB is linked to the signal recognition particle receptor FtsY. This might form the molecular basis for the potential direct involvement of FtsY in cell-division/DNA-replication processes, which is also implied by its conserved localization in an operon (in proteobacteria) with two ABC transporter proteins, FtsX and FtsE, which are involved in cell division (11, 12). The RNA helicase HrpA interacts with the antitermination protein NusA indicating an involvement of HrpA in this regulatory process, e.g. by unwinding RNA stem-loops of transcriptional terminators. The direct interaction of DNA ligase (Lig) with UvrA demonstrates a tight coupling between the first and the last step during base-excision-repair. The single-stranded DNA dependent helicase Rep is required for genome replication (13) and acts with PriC in the replication fork restart pathway, however, its specific role is unknown (14). The direct interaction of Rep with the replication helicase DnaB could indicate a concerted action of these helicases during replication fork restart.

Several proteins were newly associated with DNA-metabolism (Table 2) including a number of *Treponema* or *Spirochete* specific proteins. The protein TP0004 interacts with GyrB of the gyrase complex. Interestingly, the gene of TP0004 is located in a conserved gene cluster together with GyrA (TP0005) in spirochetes (KEGG database). This evolutionary conservation makes a functional involvement of this protein in gyrase processes very likely. DNA helicase DnaB interacts with the *Spirochetes* specific protein TP0050, which carries a phophoribosyl-transferase domain (PF00156.17, involved in nucleotide metabolism), and might link DNA replication to nucleotide metabolism either enzymatically or on the regulatory level. The *Treponema* specific protein TP0183 interacts with the DNA replication initiation protein DnaA, with SbcD, which removes DNA hairpin structures that lead to stalling of DNA replication (15), and a DNA repair helicase TP0380. Thus, TP0183 might help to reinitiate DNA replication after DNA repair mediated by SbcD and TP0380. Interestingly, the four proteins of a single (putative) operon – TP0064, TP0066, TP0067, and TP0068 – all interact with either DnaB or DnaH making an involvement of this gene cluster in DNA replication likely. TP0065 carries a N6-Adenine-specific DNA methylase signature (Prosite) and TP0067 has a high sequence similarity (46%) to the *B. burgdorferi* protein BB0195, which is a putative cell division control protein. All in all, this operon as a whole is, most probably, involved in regulation of DNA replication and cell division in *Treponema*. Finally, TP0443 has a DALR anticodon binding domain of tRNA synthetases (PF05746.5, KEGG database) and interacts with GidB, a 7-methylguanosine methyltransferase for 16S rRNA (16) with a potential involvement in cell-division. In addition, it shows an interaction with the DNA repair enzyme RecX and has a highly significant gene-neighbourhood association to another repair enzyme, RecN (combined score = 0.895) (12). Thus, TP0443 is probably involved in DNA metabolism and/or repair in *Spirochetes*.

Materials and Methods

**Datasets, orthology.** Protein interaction/association datasets were obtained from published information: *E. coli* complex purification studies (coAP/MS) (17, 18), bioinformatical predictions from the String database (12), *Campylobacter jejuni* Y2H (19), *Helicobacter pylori* Y2H (20), yeast Y2H (21-23), and yeast coAP/MS data (24). The socio-affinity-index (SAI) approach (24) was employed to extract specific (“binary”) interactions for the coAP/MS datasets. For each *E. coli* coAP/MS dataset, a “high-confidence” set with a SAI score > 5 was defined (Butland SAI, Arifuzzaman SAI). Orthology relationships between genes were taken from the MBGD (25) and the String (12) databases.

**Cloning of baits and preys and Y2H screening.** The ORF clones from McKevitt et al. (26) were transferred into compatible bait and prey vectors pLP-GADT7 (Clontech), and pLP-GBKT7Amp (a derivative of pLP-GBKT7 [Clontech] with Kan^R^ replaced by Amp^R^) or pAS1-loxP by Cre-mediated homologous recombination. All bait and prey clones were then transformed into yeast strains AH109 (baits) and Y187 (preys). All preys were arrayed and screened as described in Rajagopala et al. (27) and preys were arrayed either as quadruplicates or duplicates to ensure reproducibility, similar to the protocol described in Uetz et al. (2006) (28). Since this recent study from our lab showed that >50% of all interactions identified with our Y2H protocol can be confirmed by coIP (28), no comprehensive coIP analysis for this – much larger – dataset was attempted. We rather selected a small number of specifically interesting interactions for further verifications by coIP or overlay assay (Figure 4B and (29, 30)).

**Scoring of interactions and validation.** For selection of high-confidence datasets, all reproducible Y2H interactions were scored by two separate schemes. In the first scheme, all interactions were scored by their interaction specificity as prey protein (in-degree of directed bait->prey interaction network). Selection of the in-degree for scoring was based on our own experience (27, 28) and on the result from Bader et al. (31), who identified the degree as the only negative predictor for the quality of an interaction of a Y2H dataset. Figure S2 shows the enrichment of bioinformatically supported interactions by this scoring scheme (STRING database, combined score > 0.4). The dataset “TPA 50” was produced by defining an in-degree threshold of 50. The second, more comprehensive scoring scheme was based on logistic regression. Logistic regression is a regression model for binomially distributed dependent variables. It requires a true positive and a true negative training set and maps a number of selected predictor values to a score between 0 and 1 (with high values indicating a higher interaction reliability). **The true positive set** with 50 interactions was defined as interactions which had a homologous interaction (Blast E-value of both interacting proteins < 10^-5^) in a comprehensive interaction database combining interactions from DIP, IntAct, and the *C. jejuni* dataset (19). In addition, interactions supported by structural domain interactions from the 3DID database were considered (Supplementary Table S1). The true negative set was defined by a graph based approach. A comprehensive network of experimental interactions for *T. pallidum* was extracted from the STRING database (COG mode, experimental score > 0.15). All *T. pallidum* interactions were ranked according to the distance of their interacting proteins in this network (shortest path length in network). **A true negative set** of the same size as the true positive set was created by selecting the interactions with the highest distance. Logistic regression was done with the R package (www.r-project.org). A range of predictors was tested by taking the Akaike “information criterion” and results after cross validation taken into account (Supplementary Table S1). The number of interactions of a protein as bait (bc) and as prey (pc) and a bioinformatical association score as defined by the String database (von Mering et al., 2007) (cog_combined_score) were selected as predictors for the interaction quality. The score of each interaction can be calculated with the following formula:

.

The “TPA HCI” interaction set was defined by selecting interactions with a logistic score > 0.5. At this threshold – based on the training sets – 78% of the true interactions are classified correctly with a misclassification of 28% of the true negative interactions (= estimated false positive rate) (after 10x cross validation).

**Enrichment of String database supported interactions.** The String database contains predictions of functional protein-protein interactions (von Mering et al., 2007). These predictions are based on a number of prediction methods, including gene co-occurrence in genomes (i.e. phylogenetic profiles), gene co-expression, gene fusion events, genomic neighborhood, text mining, and experimental data. All these predictions are combined into a single score, which ranges from 0 to 1. A score of 0.4 (0.7) or greater indicates that the String database predicts a functional interaction between two proteins with medium (high) confidence. Here we asked whether interactions supported by the String database, i.e. interactions found by Y2H AND predicted by the String database, are enriched in the true interaction networks compared to randomized versions of these networks (“re-wired” networks with constant topology) (**Figure S1**). In the unfiltered *T. pallidum* interaction network (TPA all) and in the network filtered by prey-count (TPA50), interactions supported by the String database are highly enriched compared to the randomized versions of the network. As expected, no enrichment is found when predictions of low confidence (score ≥ 0.15, potential false positive predictions) are included, although the enrichment steadily increases with the confidence level of the predictions. At the high confidence levels, the enrichment in the filtered data set is higher, which shows that removing unspecific interactions preferentially retains the String database supported interactions. As a comparison, the interaction data set filtered by the comprehensive logistic regression model (TPA HCI 0.5) was included. The high enrichment of supported interactions in this set is due to the incorporation of String database predictions into the model, which was done to enrich for biological true positives (true interactions with a biological function).

Figure S1. Evaluation by String database – T. pallidum networks vs. randomized networks. The enrichment of interactions supported by the String database (Vers. 7) in three *T. pallidum* data sets (TPAall, TPA50, and TPA HCI 0.5) compared to 1000 randomized versions of these networks is shown. The enrichment is measured by a Z-score, which reflects the difference in numbers of supported interactions in the experimental and randomized networks in standard deviation units – a Z-score of 4, for example, shows that the number of interactions in the experimental network supported by the String database is 4 times the standard deviation higher than the average number for 1000 randomized versions of this network. The Z-score for the three *T. palldium* networks is calculated for four confidence levels of the String score: >0.15 (low confidence), >0.4 (medium confidence), >0.7 (high confidence), and >0.9 (highest confidence).

**Prey-count threshold vs. String database support.** For a further quality assessment of the *Treponema pallidum* two-hybrid interactions, all interactions found in the *T. pallidum* yeast-two-hybrid screen (TPA all data set) were ranked by their prey-count. The prey-count is the number of different bait proteins a prey is interacting with and thus reflects how specific the interaction pattern of a prey is. **Figure S2** shows the percentage of interactions supported by the String database (at a medium confidence level) for a given prey-count threshold. Compared to randomized versions of the network, supported interactions are enriched at lower prey-count threshold levels. This analysis shows that a) interactions supported by bioinformatical predictions are enriched in the experimentally determined data set compared to randomized versions and that b) interactions, which appear to be more specific in the Y2H assay, show a higher support by the predictions.

**Figure S2. String database support of interactions depends on prey-count threshold.** All 3684 interactions of the whole *T. pallidum* data set (X-axis) are sorted by the frequency of their preys (interactions with few rare preys are on the left while interactions involving frequent preys are on the right). This prey-count is then plotted against their serial number (the prey-count is the frequency how often a certain prey has been found, e.g. the top prey, TP0993,was found with 285 independent baits and therefore is considered unspecific. Preys TP0989 and TP0258 have been found 180 and 174 times, respectively. The red plot is the percentage of interactions at a certain preycount cut-off supported by the STRING database (combined score > 0.4). These scores provide a measure of the likelihood that two proteins are functionally associated. As a comparison, "random" shows the the average for 1000 randomized versions of the *T. pallidum* network (rewired).

**Enrichment of intra-class links in Y2H networks**. As an independent test of data quality, the enrichment of interactions within the same functional classes was assessed. Functional classes for *T. pallidum* proteins were taken from the String database (von Mering et al., 2007). **Figure S3** shows that for all three *T. pallidum* Y2H networks defined in this study (“TPA all”, “TPA50”, and “TPA HCI 0.5”): interactions within the same functional class are highly enriched compared to randomized networks. The enrichment is higher for the “TPA50” network (filtered by prey-count) compared to the whole network (“TPA all”) and, as expected, the high-confidence “TPA HCI 0.5” network has the highest enrichment (generated by a logistic regression scoring approach). This assessment, which is solely based on the functional annotations of the proteins, confirms that the whole *T. pallidum* network already shows a strong signal compared to randomized networks.

**Figure S3. Enrichment of interactions within same functional class.** Y2H networks were analyzed for interactions linking proteins of the same functional class (as defined by the String database (von Mering et al., 2007)). Each of the *T. pallidum* networks (TPA all, TPA50, and TPA HCI 0.5) was compared to 1000 randomized versions of the same network. A Z-Score was calculated, which represents the enrichment of intra-class links within the real networks compared to the randomized versions (fold standard deviation enrichment compared to random).

**Links between genomic locations (Figure 2).** Tightly connected clusters of 5 neighbouring genes were identified for the “TPA 50” network and a network of bioinformatical associations for *T. pallidum* (STRING database, protein mode, combined score > 0.7) (12). The “TPA 50” dataset (in contrast to a “TPA HCI”) was selected to exclude an influence of e.g. neighbourhood information from the STRING database, which was employed in the comprehensive filtering of the “HCI” datasets. Genomic locations with 5 neighbouring genes were selected (using a window sliding by 3 genes at a time) and the number of Y2H interactions (or association links) between all pair-wise combinations of these locations was counted. Overrepresentation of a link compared to 1000 randomized networks was assessed by calculating a Z-score, , with the number of linking interactions n, its average in 1,000 randomized networks <n_rand_>, and its standard deviation σ_rand_. Links based on at least three Y2H interactions or four bioinformatical associations, which were enriched compared to the randomized networks (Z > 2) were selected. Overlapping selected cluster links were combined by a clustering approach, which selects the genomic loci link with the highest Z-score. All interactions of the selected datasets and the identified genomic loci links were projected onto the circular chromosome of *T. pallidum* using the program Circos V0.22 by Martin Krzywinski (32).

**Associations of functional classes (Figure 3).** Associations between functional classes mediated by yeast-two-hybrid interactions and interactions derived from coAP/MS datasets were analyzed and compared for three prokaryotic and one eukaryotic species.

Yeast-two-hybrid datasets taken into consideration were the high-confidence *T. pallidum* interactome (TPA HCI 0.5) and a non-redundant union of three yeast-two-hybrid datasets for yeast ((21-23), Yeast Y2H). As CoAP/MS datasets, two complex purification studies for *E. coli* (17, 18) and one comprehensive study for yeast proteins (Gavin *et al.* (24)) were considered. The socio-affinity-index model (SAI) (24) was employed to score the affinity between a given protein-pair. Only protein-pairs with a SAI score bigger than 5 were selected. The functional classification scheme from the COG database as provided by the StringDB was employed (12), since it provides a consistent classification for bacterial as well as eukaryotic species based on orthologous gene groups.

For each association of two functional classes (i and j), the number of linking interactions n_i,k_ (excluding homodimers and reciprocal interactions) and the functional class association index fCAI_i,j_ was calculated. The fCAI represents a log-odds-ratio, which compares the odds to find the number of linking interactions in the experimental set to the odds of a random interactome model:

, with the number of total interactions n_tot_ (without homodimers and reciprocal interactions), the number of viable proteins of each class v_i_ (proteins that show at least a single interaction in the selected set), the total number of viable proteins v_tot_,, and the number of proteins which are overlapping in both classes J_i,j_.

For large sample sizes, the log of the estimated odds ratio is approximately normally distributed with the mean equal to the log of the population odds ratio and estimated variance

A test for the null hypothesis that the log-odds-ratio is 0, was based on a Z-score statistics:

Links were grouped into different significance groups with p<0.05 being the lowest significance considered.

**Extended view of *T. pallidum’s* DNA metabolism (Figure 4).** An expanded network of DNA metabolism for T. pallidum was based on a comprehensive list of DNA metabolism related proteins taken from the TIGR/CMR (www.tigr.org/CMR), the String database/COG (12), and the KEGG database (Supplementary Table S3) (33). Interaction datasets for the following species were integrated: *T. pallidum* (Y2H, TPA HCI 0.5), *C. jejuni* (Y2H, HCF data set, Parrish *et al.* (19)), *E. coli* (coAP/MS SAI, Butland *et al.* (18)), *E. coli* (coAP/MS SAI, Arifuzzaman *et al.* (17)), *B. subtilis* (Y2H, Noirot-Gros *et al.* (34), and *H. pylori* (Y2H, Rain *et al.* (20)). Predictions of bioinformatical associations between *T. pallidum* genes were taken from the STRING database (in protein mode) (12). A dataset of *E. coli* proteins localized to the bacterial nucleoid was taken from the GenoBase database (<http://ecoli.naist.jp/GB6/search.jsp>). For the transfer of interactions between species (identification of interologs), orthology relationships as defined by the MBGD database were used (default parameters except for coverage > 0.5) (33). All *T. pallidum* interactions and interologs linking two DNA metabolism related proteins were selected. In addition, interactions or interologs of DNA metabolism related proteins, which were supported by bioinformatical predictions (STRING database, protein mode, combined score > 0.4) or by at least two experimental data sets, were chosen. Finally, associated proteins, which were predicted to be involved in DNA metabolism (STRING database link with combined score > 0.8 to DNA metabolism related proteins), localized to the nucleoid in *E. coli*, or had an additional evidence associated with it (Table 2) were included. Network visualization was done with the Cytoscape software (35).

A number of these selected interactions were re-tested by co-immunoprecipitation as described in Rajagopala *et al.* (2). Briefly, the respective genes were cloned into *E. coli* expression vectors based on the pBAD series (36) using a Cre-recombinase based cloning system (see above). One gene was cloned into a modified pBAD-33 vector with added loxP-site and HA-tag, the other gene was cloned into a modified pBAD-24 with added loxP-site and Myc-tag. L-arabinose induced protein expression was conducted in *E. coli* TOP10 cells (Invitrogen, CA, USA). Co-immunoprecipitation was done with a monoclonal anti-Myc antibody (clone 9E10, Santa Cruz) and Western Blot with the same antibody and a polyclonal anti-HA antibody (Rockland).

**Functional classification by guilt-by-association approach**. Functional annotations of proteins were taken from the STRING database (12). The number of interactions of each protein to each functional class was counted (excluding homodimers). The obtained number of interactions was compared to its distribution in 1000 randomized networks. Only protein/class links with a Z-score > 3 and more than one interaction were considered. For 28 proteins with a known function, a functional prediction was made, of which 43% were correct. Misclassification can be explained by cross-talk between functional complexes, and thus only functional predictions supported by additional evidences were considered (Table 2).

**Prediction of interactions (interologs) for other species.** For each interaction in our TPA HCI set we predicted an interaction (interolog) for a target species if both interacting partners had an ortholog (STRING COG orthology) in the same. In addition, we only retained orthologs whose protein sequence was covered by more than 50% of the conserved region of the respective orthologous protein family. Note: Due to paralogs, one of our interactions might predict several interactions in the target species.

**References**

1. Edwards, A. M., Kus, B., Jansen, R., Greenbaum, D., Greenblatt, J., & Gerstein, M. (2002) *Trends Genet* **18,** 529-536.

2. Rajagopala, S. V., Titz, B., Goll, J., Parrish, J. R., Wohlbold, K., McKevitt, M. T., Palzkill, T., Mori, H., Finley, R. L., & Uetz, P. (2007 (in press)) *Molecular Systems Biology* **3**.

3. Deaconescu, A. M., Savery, N., & Darst, S. A. (2007) *Current opinion in structural biology* **17,** 96-102.

4. Trautinger, B. W., Jaktaji, R. P., Rusakova, E., & Lloyd, R. G. (2005) *Molecular cell* **19,** 247-258.

5. Sikder, D., Unniraman, S., Bhaduri, T., & Nagaraja, V. (2001) *Journal of molecular biology* **306,** 669-679.

6. Harmon, F. G. & Kowalczykowski, S. C. (1998) *Genes & development* **12,** 1134-1144.

7. Harmon, F. G. & Kowalczykowski, S. C. (2001) *The Journal of biological chemistry* **276,** 232-243.

8. Umezu, K. & Nakayama, H. (1993) *Journal of molecular biology* **230,** 1145-1150.

9. Boubrik, F. & Rouviere-Yaniv, J. (1995) *Proc Natl Acad Sci U S A* **92,** 3958-3962.

10. Huisman, O., Faelen, M., Girard, D., Jaffe, A., Toussaint, A., & Rouviere-Yaniv, J. (1989) *J Bacteriol* **171,** 3704-3712.

11. Gill, D. R. & Salmond, G. P. (1987) *Mol Gen Genet* **210,** 504-508.

12. von Mering, C., Jensen, L. J., Kuhn, M., Chaffron, S., Doerks, T., Kruger, B., Snel, B., & Bork, P. (2007) *Nucleic Acids Res* **35,** D358-362.

13. Takahashi, S., Hours, C., Chu, A., & Denhardt, D. T. (1979) *Canadian journal of biochemistry* **57,** 855-866.

14. Heller, R. C. & Marians, K. J. (2005) *Molecular cell* **17,** 733-743.

15. Connelly, J. C., Kirkham, L. A., & Leach, D. R. (1998) *Proc Natl Acad Sci U S A* **95,** 7969-7974.

16. Okamoto, S., Tamaru, A., Nakajima, C., Nishimura, K., Tanaka, Y., Tokuyama, S., Suzuki, Y., & Ochi, K. (2007) *Molecular microbiology* **63,** 1096-1106.

17. Arifuzzaman, M., Maeda, M., Itoh, A., Nishikata, K., Takita, C., Saito, R., Ara, T., Nakahigashi, K., Huang, H. C., Hirai, A.*, et al.* (2006) *Genome Res* **16,** 686-691.

18. Butland, G., Peregrin-Alvarez, J. M., Li, J., Yang, W., Yang, X., Canadien, V., Starostine, A., Richards, D., Beattie, B., Krogan, N.*, et al.* (2005) *Nature* **433,** 531-537.

19. Parrish, J. R., Yu, J., Liu, G., Hines, J. A., Chan, J. E., Mangiola, B. A., Zhang, H., Pacifico, S., Fotouhi, F., Dirita, V. J.*, et al.* (2007) *Genome Biol* **8,** R130.

20. Rain, J. C., Selig, L., De Reuse, H., Battaglia, V., Reverdy, C., Simon, S., Lenzen, G., Petel, F., Wojcik, J., Schachter, V.*, et al.* (2001) *Nature* **409,** 211-215.

21. Hazbun, T. R., Malmstrom, L., Anderson, S., Graczyk, B. J., Fox, B., Riffle, M., Sundin, B. A., Aranda, J. D., McDonald, W. H., Chiu, C. H.*, et al.* (2003) *Molecular cell* **12,** 1353-1365.

22. Ito, T., Chiba, T., Ozawa, R., Yoshida, M., Hattori, M., & Sakaki, Y. (2001) *Proc Natl Acad Sci U S A* **98,** 4569-4574.

23. Uetz, P., Giot, L., Cagney, G., Mansfield, T. A., Judson, R. S., Knight, J. R., Lockshon, D., Narayan, V., Srinivasan, M., Pochart, P.*, et al.* (2000) *Nature* **403,** 623-627.

24. Gavin, A. C., Aloy, P., Grandi, P., Krause, R., Boesche, M., Marzioch, M., Rau, C., Jensen, L. J., Bastuck, S., Dumpelfeld, B.*, et al.* (2006) *Nature* **440,** 631-636.

25. Uchiyama, I. (2003) *Nucleic Acids Res* **31,** 58-62.

26. McKevitt, M., Patel, K., Smajs, D., Marsh, M., McLoughlin, M., Norris, S. J., Weinstock, G. M., & Palzkill, T. (2003) *Genome Res* **13,** 1665-1674.

27. Rajagopala, S. V., Titz, B., & Uetz, P. (2007) in *Methods in Microbiology*, pp. 139-163,701.

28. Uetz, P., Dong, Y. A., Zeretzke, C., Atzler, C., Baiker, A., Berger, B., Rajagopala, S. V., Roupelieva, M., Rose, D., Fossum, E.*, et al.* (2006) *Science* **311,** 239-242.

29. Rajagopala, S. V., Titz, B., Goll, J., Parrish, J. R., Wohlbold, K., McKevitt, M. T., Palzkill, T., Mori, H., Finley, R. L., Jr., & Uetz, P. (2007) *Mol Syst Biol* **3,** 128.

30. Titz, B., Rajagopala, S. V., Ester, C., Hauser, R., & Uetz, P. (2006) *J Bacteriol* **188,** 7700-7706.

31. Bader, J. S., Chaudhuri, A., Rothberg, J. M., & Chant, J. (2004) *Nature Biotechnology* **22,** 78-85.

32. Krzywinski, M. (2007) http://mkweb.bcgsc.ca/circos/.

33. Uchiyama, I. (2007) *Nucleic Acids Res* **35,** D343-346.

34. Noirot-Gros, M. F., Dervyn, E., Wu, L. J., Mervelet, P., Errington, J., Ehrlich, S. D., & Noirot, P. (2002) *Proc Natl Acad Sci U S A* **99,** 8342-8347.

35. Shannon, P., Markiel, A., Ozier, O., Baliga, N. S., Wang, J. T., Ramage, D., Amin, N., Schwikowski, B., & Ideker, T. (2003) *Genome Res* **13,** 2498-2504.

36. Guzman, L. M., Belin, D., Carson, M. J., & Beckwith, J. (1995) *J Bacteriol* **177,** 4121-4130.
