# Supplementary figures and images for: The Binary Protein Interactome of Treponema pallidum – The Syphilis Spirochete
Source: PLoS One. 2008 May 28;3(5):e2292. doi: 10.1371/journal.pone.0002292 (PMC2386257; doi:10.1371/journal.pone.0002292)

Figure 5

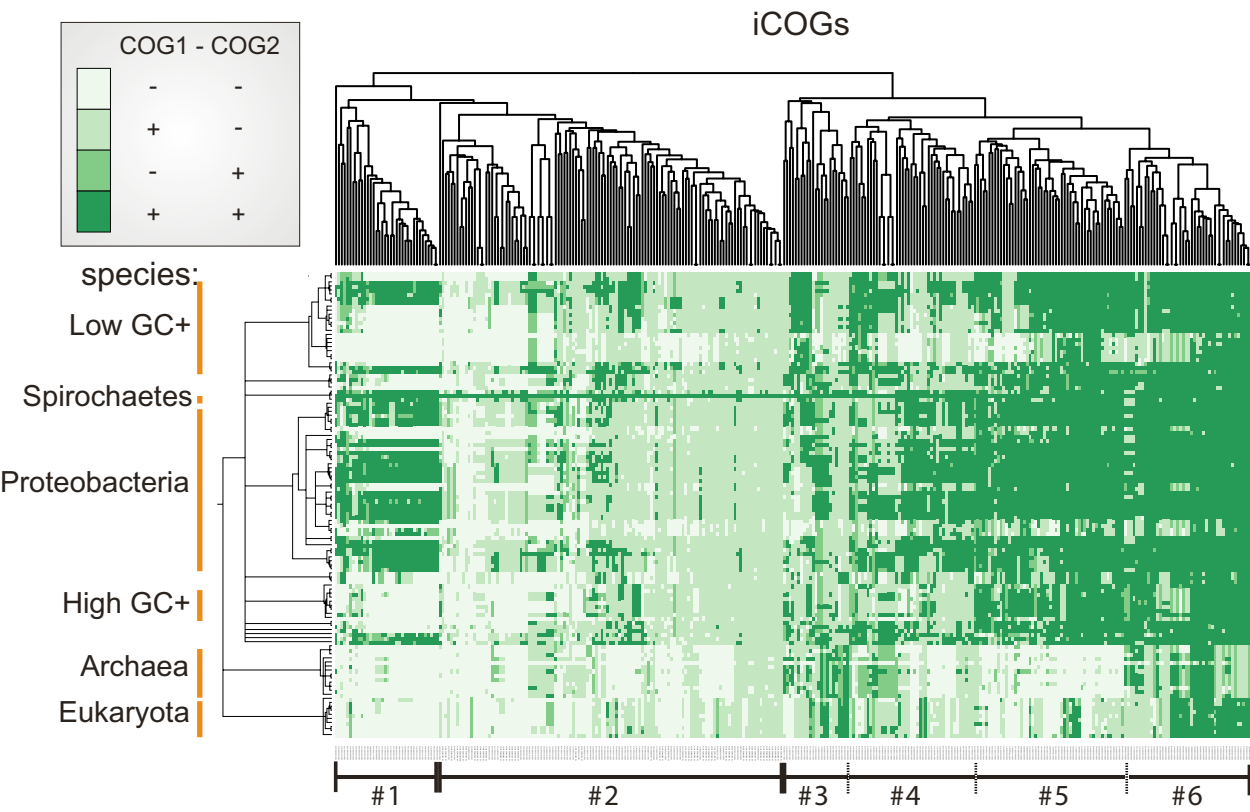

Supplement: Figure S1 — Interacting clusters of orthologous groups (“iCOG”) show phylogenetically conserved interaction patterns. Each row of the shown profile corresponds to a species and each column corresponds to a pair of interacting protein families (i.e. iCOG), for which an interaction was found in the high-confidence T. pallidum data set. The protein families were defined based on the “cluster of orthologous genes” approach (COG) (see methods). With this, the profile shows for each interaction of the T. pallidum data set whether both interacting proteins, only one interacting protein or none of the interacting proteins are conserved in a given species (given row). For each species from the shown taxonomy (y-axis) and each iCOG, a conservation value is shown in the matrix. This conservation value indicates whether both COGs are conserved/absent in a given species or whether only one or the other COG is conserved (see left upper corner for color key). Overall, three distinct conservation regions are visible in the clustered matrix: #1, #2, and region #3-#6, which we subdivided somewhat arbitrarily into individual clusters #3-#6 with increasing conservation from left to right (note branches on tree above). (0.14 MB PDF) [file pone.0002292.s007.pdf]
